# Positive leadership and health-related long-term outcomes among hospital nurses: a cross-sectional study

**DOI:** 10.3389/frhs.2026.1789258

**Published:** 2026-05-22

**Authors:** Christoph Golz, Mirjam Körner, Fabienne Josefine Renggli, Kai-Uwe Schmitt

**Affiliations:** 1 Applied Research & Development in Nursing, School of Health Professions, Bern University of Applied Sciences, Bern, Switzerland; 2 Institute for Collaborative Practice and Leadership in Health Care, School of Health Professions, Bern University of Applied Sciences, Bern, Switzerland; 3 Academic-Practice-Partnership Between Bern University of Applied Sciences and Bern University Hospital, School of Health Professions, Bern University of Applied Sciences, Bern, Switzerland

**Keywords:** cross-sectional, healthcare, leadership, nurse, PERMA-Lead

## Abstract

**Background:**

Nurses face high job demands that increase the risk of burnout, reduced job satisfaction, and intention to leave the profession. Leadership is a key resource for improving working conditions in healthcare, yet research has largely focused on deficit-oriented approaches. Positive leadership, grounded in positive psychology, aims to actively promote well-being and flourishing. The PERMA-Lead model operationalizes this approach through five dimensions: Positive Emotions, Engagement, Relationships, Meaning, and Accomplishment. Evidence on PERMA-Lead in nursing contexts remains limited.

**Aim:**

This study examined perceptions of positive leadership among nurses and nurse managers and investigated associations between PERMA-Lead and health-related long-term outcomes.

**Methods:**

A cross-sectional survey was conducted in an acute care hospital in the German-speaking part of Switzerland. Nurses and nurse managers (*N* = 211; response rate 23%) completed an online questionnaire including the PERMA-Lead Profiler and validated Copenhagen Psychosocial Questionnaire scales assessing job satisfaction, burnout symptoms, and intention to leave the profession. Group differences were analyzed using Welch's *t*-tests. Associations were examined using multiple linear regression models with robust standard errors, controlling for established workplace factors. Sensitivity analyses using more parsimonious models were conducted to assess the influence of model specification. Missing data were handled using multiple imputation.

**Results:**

PERMA-Lead levels were high across all dimensions (means >78 on a 0–100 scale). Managers rated their leadership higher than non-management staff (*p* = .002). Higher PERMA-Lead was consistently associated with lower intention to leave the profession (*β* = −0.64, *p* = .002; R^2^ = .53). In contrast, associations with job satisfaction depended on model specification, with no significant association observed in the fully adjusted model (*β* = 0.00, *p* = .95), whereas no association was found for burnout symptoms (*β* = 0.11, *p* = .52). Burnout symptoms were associated with work–private life conflict and insecurity of the working environment, while job satisfaction was mainly associated with core work resources.

**Conclusions:**

Positive leadership appears relevant for nurse retention. In contrast, its association with job satisfaction is less clear and depends on model specification, while no association was observed with burnout symptoms. Leadership may need to be complemented by structural improvements in working conditions.

## 1 Introduction

Nurses form the backbone of healthcare systems worldwide, yet they are exposed to numerous stressors that threaten their health and job retention (1). Internationally, nursing shortages are a persistent challenge, exacerbated by premature exits from the profession (2, 3). High job demands (quantitative or emotional), low influence at work, and work-privacy conflict are known work-related stressors, among various others, that contribute to burnout, intention to leave, and decreased job satisfaction (4, 5). Leadership has been identified as a critical factor in mitigating negative outcomes and fostering sustainable working conditions in healthcare (6–8) In particular, a substantial body of research highlights leadership as a key determinant of nurses' job satisfaction, with leadership quality positively associated with both nurses' intention to stay and job satisfaction (5, 9, 10), and evidence consistently indicates that supportive and fair leadership reduces stress, burnout, and presenteeism while strengthening job satisfaction and retention (5, 6). Similarly, positive associations between person-centered care and outcomes such as job satisfaction, lower burnout symptoms, a better psychosocial work environment, and reduced intention to leave were significant in several cross-sectional studies, whereas findings from longitudinal studies have been less conclusive (11). Conversely, poor leadership can exacerbate stressors such as role ambiguity, contributing to turnover intentions and deteriorating well-being. Thus, leadership has been seen as crucial in interventions aimed at improving nurses' working conditions (12) and various studies have shown that leadership fosters participation (13, 14).

Leadership has therefore emerged as a central resource for shaping healthy and attractive work environments in nursing. However, much of the existing evidence focuses on leadership as a protective factor that reduces strain and prevents adverse outcomes. This deficit-oriented view is essential, yet it overlooks leadership's potential to build well-being, engagement, and personal growth actively. Against this background, approaches that move beyond mere stress mitigation toward the systematic promotion of positive experiences at work have gained attention (6, 15, 16).

Positive leadership, grounded in positive psychology, aims not only to prevent stress and ill health but also to promote well-being and flourishing at work (17, 18). To operationalize this approach, frameworks are needed that specify how leaders can systematically foster positive experiences in their teams. The PERMA framework offers such a foundation by identifying five essential building blocks of thriving. Adapted to leadership contexts, the PERMA framework—Positive Emotions, Engagement, Relationships, Meaning, and Accomplishment—provides a practical structure for leaders to foster well-being in their teams (19). The PERMA-Lead model posits that leaders who cultivate these five domains among their staff enhance well-being, resilience, and organizational commitment (19).

While most research focuses on transformational or health-oriented leadership, no studies have applied the PERMA-Lead framework in nursing contexts. Positive leadership emphasizes not only avoiding adverse outcomes but also systematically developing positive states such as meaning, growth, and strong social bonds. These aspects are especially relevant for nurses, given their emotionally demanding work and reliance on teamwork (20). Moreover, the PERMA-Lead approach conceptually aligns with person-centeredness, a foundational value in nursing practice (21). Both frameworks emphasize relational care, shared decision-making, and the creation of healthful work environments. Given these conceptual overlaps, we regard PERMA-Lead and person-centeredness as complementary perspectives. PERMA-Lead highlights leadership behaviors that foster the types of flourishing experiences that person-centered approaches seek to promote (22, 23).

This study investigated PERMA-Lead in nursing leadership in a German-speaking hospital in Switzerland. It (1) describes current levels of positive leadership from employee and manager perspectives, (2) compares these perspectives, and (3) analyzes associations between PERMA-Lead and long-term outcomes (job satisfaction, burnout symptoms, intention to leave), while accounting for established relevant factors.

### 1.1 Design

The study uses a cross-sectional survey design within the STRAIN 2.0 project. In the STRAIN 2.0 project, health organizations in Switzerland are supported in monitoring working conditions and evaluating measures aimed at improving them (24).

We adhered to the STROBE reporting guidelines for cross-sectional studies (25). The checklist can be found as Supplementary File A.

## 2 Materials and methods

### 2.1 Recruitment

The STRAIN 2.0 project is based on a convenience sample of Swiss health organizations. One acute hospital in the German-speaking part of Switzerland has decided to implement PERMA-Lead as a promising approach to improving the working conditions of its health professionals.

### 2.2 Data collection

Participants included registered nurses and nurse managers. All eligible staff (*N* = 932) were invited to participate voluntarily. Respondents were categorized as management or non-management (staff nurses without leadership responsibility). For data collection, the nursing director was responsible for distributing the link of the online questionnaire. Data were collected between April and June 2025 using an online questionnaire.

#### 2.2.1 Instruments

For this study, a questionnaire was developed based on the “Model of causes and consequences of work-related stress” (14, 26, 27). In this model, the outcomes intention to leave the profession, job satisfaction, and burnout symptoms can be allocated to long-term consequences and PERMA-Lead as an added predictor to a list of further known predictors.

The following valid and reliable scales from the Copenhagen Psychosocial Questionnaire (COPSOQ) (28) were used to measure three known consequences of work-related stress: Burnout-Symptoms (*n* = 3) and Job Satisfaction (*n* = 6). Intention to leave the profession was measured using a well-established single item also from the COPSOQ “In the past 12 months, how often have you thought about giving up your profession?” All item responses of the COPSOQ were scored on a five-point Likert scale.

With the PERMA-Lead Profiler (*n* = 15), we assessed leadership behaviors across five dimensions: Positive Emotions, Engagement, Relationships, Meaning, and Accomplishment from the employee and the management perspective (19). The Profiler is under license and publication of the items is limited to example items per dimension:

- Positive Emotions: I contribute to the fact that my employees feel comfortable at the workplace.
- Engagement: I deliberately give my employees tasks that suit their individual strengths.
- Relationships: I make sure that the members of my team support each other.
- Meaning: I contribute to my employees experiencing meaning in their work.
- Accomplishment: I share my employees' joy when they have reached a (partial) goal, and I also tell them that.

**Figure 1:**
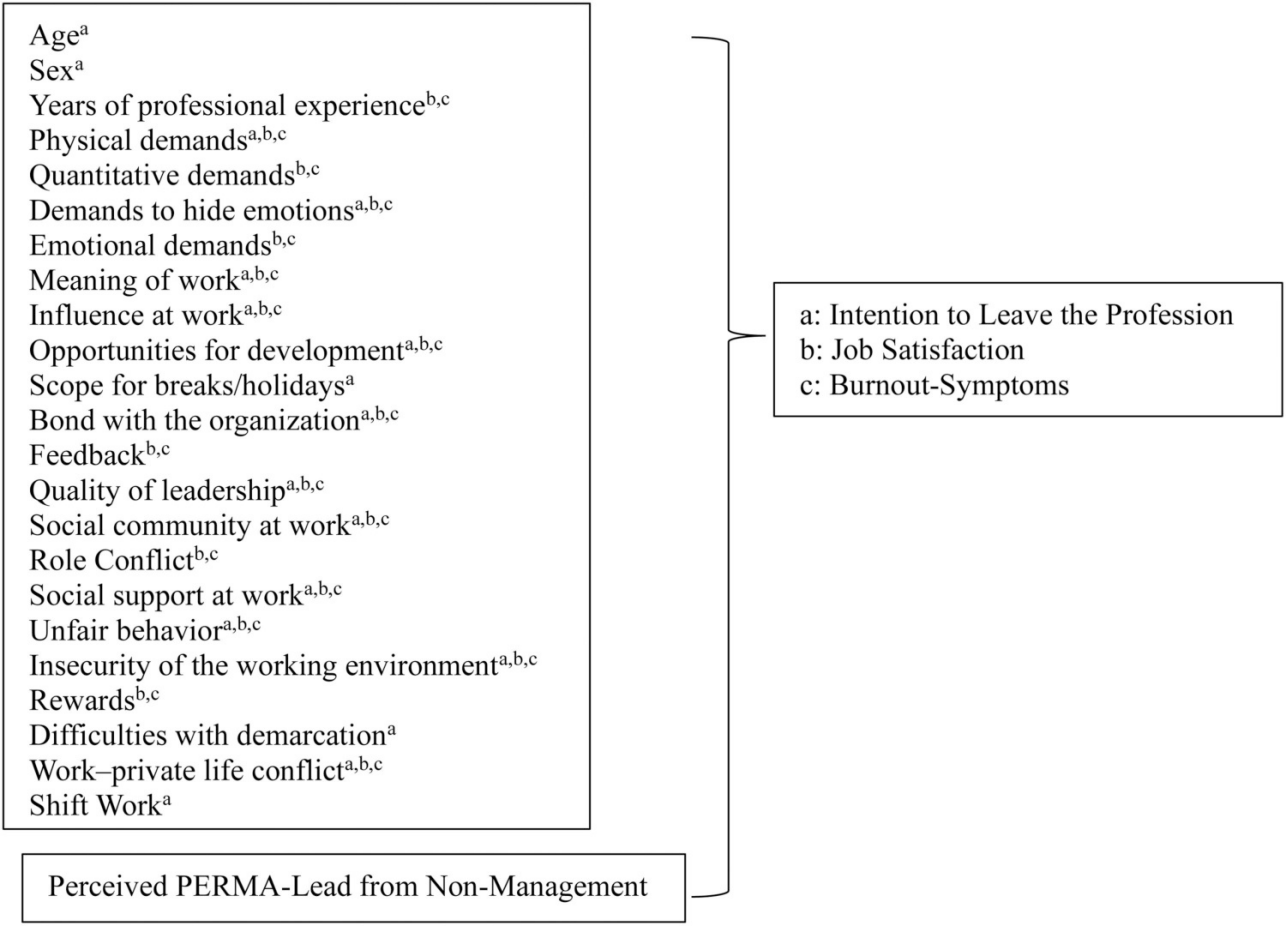
Predictors and outcomes in the three regression models.

The selection of further predictors was based on previous evidence from Peter et al. (5), who identified relevant workplace and psychosocial determinants of these outcomes in a large Swiss sample. The predictors included in the analysis are presented in Figure 1. These predictors capture distinct but related dimensions of the work environment, including functional aspects of leadership (e.g., planning and conflict management), social support, team-related resources, and the perceived meaning of work and are all scales from the COPSOQ.

Scores were transformed to a 0–100 scale, with higher scores indicating stronger intention, job satisfaction, burnout symptoms, and positive leadership. Consistent with previous COPSOQ-based research (5), the single items *Intention to leave the profession, Unfair behavior and Rewards* are treated as approximately continuous after transformation to a 0–100 scale, allowing for the use of linear regression models.

Internal consistency of the multi-item COPSOQ scales and the PERMA-Lead Profiler for the study sample was assessed using Cronbach's alpha. For the COPSOQ scales, internal consistency ranged from moderate to excellent in the present sample (Cronbach's *α* = 0.60–0.90). The PERMA-Lead Profiler showed very high internal consistency (Cronbach's *α* = 0.98).

### 2.3 Data analysis

Data were analyzed using R (version 4.5.2, R Foundation for statistical Computing) (29). Mean scores were computed for the COPSOQ scales and for the PERMA-Lead Profiler. For COPSOQ scales, mean scores were calculated only if at least half of the items of each scale were answered. Descriptive statistics were produced for all variables, including means, standard deviations and observed ranges.

Model assumptions, including linearity, normality of residuals, and homoscedasticity, were assessed. Assumption checks indicated that one group deviated from normality (Shapiro–Wilk *p* < .001) and variances were unequal (Levene's test *p* = .001). Therefore, group differences in PERMA scores were examined using Welch's *t*-test, which does not assume equal variances and is robust to violations of normality (30).

Associations between PERMA-Lead and the three outcomes intention to leave the profession, job satisfaction and burnout symptoms were analyzed using multiple linear regressions while controlling for established predictors.

Several covariates were conceptually related to PERMA-Lead but were initially included to assess its incremental contribution beyond established workplace predictors. To evaluate potential conceptual overlap, we examined correlations among the main predictors. We then conducted sensitivity analyses using more parsimonious models that excluded variables theoretically proximate to PERMA-Lead (Table 1). These variables were removed based on conceptual rather than statistical considerations to assess the extent to which estimates of PERMA-Lead were affected by overlap and potential overadjustment. The parsimonious models were not intended for variable selection or prediction, but to evaluate the stability of associations across theoretically informed specifications. An overview of excluded variables and their conceptual rationale is provided in Table 1, with a detailed description in Supplementary File B.

**Table 1:** Conceptually proximal variables excluded from parsimonious models.

Variable	Conceptual rationale for exclusion
Meaning of work	Reflects PERMA dimension meaning
Opportunities for development	Aligns with engagement and accomplishment
Bond with the organization	Reflects relational/motivational attachment
Feedback	Core leadership behaviour
Quality of Leadership	Strong conceptual overlap with PERMA-Lead
Social community at work	Leadership-shaped relational climate
Social support at work	Leadership-driven support processes
Rewards	Reflects recognition and appreciation

Missing data were addressed using multiple imputations with the mice package. Predictive mean matching was applied with twenty imputations and fifty iterations. All variables included in the regression models were also used as predictors in the imputation models to preserve the data's multivariate structure. After imputation, the same regression model was estimated in each dataset, and the estimates were pooled using Rubin's rules. Descriptive statistics and missingness patterns were based on the original (non-imputed) dataset, whereas regression analyses were conducted using the multiply imputed datasets. The extent of missing data for all variables is reported as percentages in Supplementary File C, with no variable exceeding 15% missingness.

Regression assumptions were checked on the first imputed dataset. Linearity and normality of residuals were acceptable and variance inflation factors indicated no problematic multicollinearity. The Breusch Pagan test indicated heteroscedasticity across models; therefore, all regression coefficients were estimated with heteroscedasticity-consistent HC3 robust standard errors in each imputed dataset prior to pooling. In the final pooled model, statistical significance was defined as *p* < 0.05.

As an additional sensitivity check for the handling of missing data, complete-case analyses of the fully adjusted models were conducted and compared with the corresponding multiply imputed models.

Manager self-ratings of PERMA-Lead were collected descriptively but were not used in regression analyses due to the very small number of managers (*n* = 34), which is insufficient for multivariable modeling. All regression models, therefore, focused on employee-reported leadership perceptions, which were available at an adequate sample size.

### 2.4 Ethics

The responsible Swiss ethical board in Bern (Kantonale Ethikkommission Bern) confirmed that approval for the ’STRAIN 2.0' study is deemed unnecessary according to national legislation and does not fall under the Swiss Federal Act on Research Involving Human Beings (reference number: Req-2021-01162). The study was conducted in accordance with the Declaration of Helsinki. All health employees of the participating organizations were informed about the aim, procedures, risks, benefits, anonymity of data, and alternatives for participating in the study via online announcement so that they could make an informed decision about participation. Informed consent was obtained, and participants were again informed on the first page of the questionnaire about the aim, procedure, and possible risks and had to actively give their consent (written, using a checkbox) to continue the questionnaire. The study was carried out on a voluntary basis for all participating organizations and health professionals; all participants were free to stop filling out the questionnaire at any time and had the opportunity to contact the research team via hotline if they had any questions.

## 3 Results

The study sample comprised 211 participants (response rate 23 percent). Most participants were female (92 percent), with a mean age of 40 years (SD = 12.6). On average, they had 18.5 years (SD = 12) of professional experience. Detailed sample characteristics are shown in Table 2.

**Table 2:** Sample characteristics.

Variable	Mean (SD)	*N* (%)
Age	40.0 (12.6)	
Sex		
Female		194 (92)
Male		17 (8)
Education		
No education		7 (3)
Secondary II		38 (18)
Tertiary B		83 (39)
Bachelor of Science		72 (34)
Master of Science		9 (4)
Missing		2 (1)
Management		
No		177 (84)
Yes		34 (16)
Years of professional experience	18.5 (12)	

### 3.1 PERMA-Lead

Both employee (*N* = 177) and manager (*N* = 34) ratings indicated consistently high levels of perceived positive leadership, with mean values above 78 on the 0–100 scale. Across dimensions, Relationships and Accomplishment emerged as particularly strong, while Positive Emotions and Engagement showed more variability (Table 3).

**Table 3:** PERMA-Lead descriptives.

PERMA-Lead dimension	Non-Management		Management	
	Mean	SD	Mean	SD
Positive Emotions	81.2	17.2	85.3	10.4
Engagement	78.2	20.3	84.0	11.8
Relationships	82.8	18.6	91.0	7.4
Meaning	80.7	19.4	89.4	7.8
Accomplishment	83.2	20.3	89.3	9.2
Total	81.4	17.5	88.1	7.7

### 3.2 Management vs. Non-Management

Managers rated their own leadership consistently higher than employees did. A Welch *t*-test confirmed this difference, t(121.1) = −3.14, *p* = .002. Non-management staff reported lower PERMA-Lead scores (M = 81.5) than managers (M = 87.5), with a 95 percent confidence interval for the mean difference of −9.86 to −2.24. This indicates a difference between managers' self-ratings and employees' perceptions.

### 3.3 Multiple linear regressions

The results of the three regression models are summarized in Table 4. Correlations among predictors were moderate (*r* = .30–.60). In particular, PERMA-Lead showed a strong correlation with leadership quality (*r* = .73), indicating substantial conceptual overlap, although not complete redundancy (see Supplementary File D).

**Table 4:** Pooled estimates of linear regression models with heteroscedasticity-consistent standard errors.

	Intention to leave the profession				Job satisfaction				Burnout-Symptoms			
Predictor	*β*	SE	95% CI	*p*	β	SE	95% CI	*p*	β	SE	95% CI	*p*
Intercept	52.94	30.61	−7.10–112.97	.103	35.98	8.76	18.68–53.27	.001	7.33	21.24	−34.35–48.99	.731
PERMA−Lead	−0.64	0.21	−1.05–−0.23	.**002**	0.00	0.07	−0.13–0.14	.954	0.11	0.17	−0.23–0.46	.515
Age	−0.33	0.33	−0.98–0.33	.323					−0.07	0.15	−0.37–0.22	.627
Years of professional experience	0.03	0.36	−0.68–0.73	.938	−0.04	0.06	−0.16–0.09	.565				
Sex: Male	−3.06	8.15	−19.03–12.92	.696					−14.73	5.45	−25.54–−3.92	.**008**
Emotional demands	0.09	0.15	−0.20–0.38	.537	−0.01	0.05	−0.11–0.10	.882				
Physical Demands					−0.08	0.04	−0.15–−0.00	.**043**	0.12	0.08	−0.04–0.29	.145
Quantitative demands	0.10	0.17	−0.23–0.43	.560	−0.04	0.05	−0.14–0.07	.480				
Demands to hide emotions	0.08	0.12	−0.15–0.31	.500	−0.04	0.03	−0.11–0.02	.220	0.15	0.08	−0.01–0.32	.076
Meaning of work	−0.09	0.17	−0.43–0.25	.601	0.02	0.05	−0.09–0.12	.765	−0.22	0.14	−0.48–0.04	.112
Influence at work	−0.31	0.12	−0.55–−0.07	.**013**	0.03	0.04	−0.04–0.11	.339	0.00	0.1	−0.20–0.19	.971
Opportunities for development	−0.03	0.24	−0.50–0.45	.902	0.10	0.06	−0.03–0.22	.140	−0.02	0.16	−0.34–0.30	.909
Scope for breaks/holidays									0.06	0.09	−0.12–0.25	.504
Bond with the organization	−0.18	0.16	−0.50–0.13	.252	0.13	0.05	0.04–0.22	.**007**	0.03	0.12	−0.22–0.29	.808
Feedback					−0.03	0.04	−0.11–0.04	.376				
Quality of leadership	0.24	0.19	−0.13–0.62	.207	0.10	0.07	−0.03–0.23	.131	0.00	0.16	−0.31–0.31	.994
Social community at work	0.18	0.15	−0.12–0.49	.243	0.16	0.05	0.06–0.27	.**003**	0.16	0.14	−0.11–0.43	.238
Role conflict	0.00	0.10	−0.20–0.20	.995	−0.01	0.03	−0.08–0.06	.770				
Social support at work					0.09	0.07	−0.05–0.23	.198	0.08	0.17	−0.25–0.41	.623
Unfair behaviour	−0.07	0.11	−0.28–0.14	.513	−0.02	0.04	−0.10–0.06	.585	0.03	0.09	−0.15–0.21	.766
Insecurity of the working environment	0.27	0.09	0.08–0.45	.**004**	−0.06	0.03	−0.13–0.00	.052	0.22	0.08	0.06–0.38	.**009**
Rewards	0.28	0.10	0.07–0.49	.**009**	0.09	0.04	0.01–0.16	.**021**	0.04	0.09	−0.15–0.23	.689
Difficulties with demarcation	−0.03	0.09	−0.21–0.16	.749					−0.1	0.09	−0.28–0.08	.265
Work–private life conflict	0.19	0.14	−0.09–0.47	.180	−0.06	0.04	−0.14–0.02	.115	0.44	0.11	0.23–0.66	.**001**
Shift work	−7.39	4.45	−16.13–1.35	.097								

Regression coefficients (β), standard errors (SE), confidence intervals (CI), and *p*-values. Bold values indicate statistically significant associations (*p* < .05). Estimates are based on pooled results from multiple imputation (*m* = 20).

The first pooled linear regression model examined predictors of intention to leave the profession. The model was statistically significant, F(20, 129) = 7.24, *p* < .001, and explained 53% of the variance (R^2^ = .529, 95% CI.403 to.638). PERMA-Lead emerged as a significant negative predictor, indicating that nurses who perceived higher levels of positive leadership reported a lower intention to leave their profession. Beyond PERMA-Lead, several work-related characteristics were associated with turnover intentions, including lower influence at work, greater insecurity in the work environment, and lower rewards.

The second pooled regression model focused on job satisfaction, F(19, 130) = 22.34, *p* < .001, and accounted for a large proportion of variance (R^2^ = .766, 95 percent CI.682 to.830). In the fully adjusted model, PERMA-Lead was not significantly associated with job satisfaction after controlling for psychosocial and workplace covariates (see sensitivity analyses below). Instead, job satisfaction was mainly associated with core work resources such as social community at work, rewards, and bond with the organization.

The third regression model examined burnout symptoms, F(19, 130) = 7.41, *p* < .001, explaining 52 percent of the variance (R^2^ = .520, 95 percent CI.387 to.636). In contrast to intention to leave, PERMA-Lead was not significantly associated with burnout symptoms in the fully adjusted model. Burnout symptoms were instead more strongly associated with stress-related working conditions, including insecurity of the working environment and work–private life conflict. Male nurses reported fewer burnout symptoms than female nurses.

Sensitivity analyses using more parsimonious models excluding conceptually proximal variables (e.g., leadership quality, social support, and meaning of work) provided a more differentiated picture. The association between PERMA-Lead and intention to leave the profession remained consistent in direction and statistically significant across model specifications. In contrast, the association with job satisfaction changed across specifications and became stronger when conceptually overlapping variables were excluded, indicating that this finding is sensitive to model specification. No substantial changes were observed for burnout symptoms, which remained non-significant across models. Table 5 presents a direct comparison of PERMA-Lead coefficients across model specifications. Full model outputs of the parsimonious models are provided in Supplementary File E.

**Table 5:** Comparison of PERMA-lead coefficients across fully adjusted and parsimonious models.

Outcome	Model specification	β (PERMA-Lead)	95% CI	*p*-value
Intention to leave	Fully adjusted	−0.64	−1.05–−0.23	.002
	Parsimonious	−0.36	−0.67–−0.05	.024
Job satisfaction	Fully adjusted	0.00	−0.13–0.14	.954
	Parsimonious	0.24	0.12–0.36	.001
Burnout symptoms	Fully adjusted	0.11	−0.23–0.46	.515
	Parsimonious	0.20	−0.07–0.47	.152

Regression coefficients for PERMA-Lead (β), confidence intervals (CI), and *p*-values from sensitivity analyses.

Complete-case analyses of the fully adjusted models yielded similar effect sizes and consistent significance patterns across all models. The association between PERMA-Lead and intention to leave remained similar (*β* = −0.61 vs. −0.64 in the imputed model), while no associations were observed for job satisfaction (*β* = 0.03, *p* = .676) or burnout (*β* = 0.14, *p* = .435) in the fully adjusted complete-case models.

## 4 Discussion

This study investigated perceptions of positive leadership among nurses and nurse managers and examined associations with health-related outcomes in a Swiss acute care hospital. Overall, three main findings emerged. First, PERMA-Lead scores were high across groups and dimensions, suggesting that many elements of positive leadership are already present in this setting. Second, managers rated their own leadership more positively than employees did, indicating a difference between manager self-ratings and employee perceptions. Third, PERMA-Lead remained significantly associated with intention to leave the profession across model specifications, whereas findings for job satisfaction depended on model specification and findings for burnout symptoms were consistently non-significant.

The generally high PERMA-Lead scores align with previous work showing that nurses often experience strong meaning in their work, close relationships with colleagues, and a high sense of professional purpose, even under demanding conditions. Positive leadership approaches that emphasize strengths, constructive feedback, and shared purpose are increasingly promoted to create thriving work environments in healthcare. Studies on engaging and transformational leadership styles have consistently linked such behaviors to higher work engagement, better team climate, and stronger organizational commitment among nurses (31, 32). At the same time, the high PERMA-Lead scores in our study may partly reflect response tendencies such as social desirability and self-enhancement, which are well-documented in leadership research. As summarized in Cummings et al.'s large systematic review, most leadership studies rely heavily on self-reported outcomes rather than observed or independently rated behaviors, introducing potential upward bias in leadership perceptions (14). In addition, the uniformly high scores may indicate ceiling effects and restricted variability, which can attenuate associations with outcome variables.

The finding that managers evaluated their own leadership more positively than non-management staff nurses did should be interpreted with caution, as the study design does not allow matching manager self-ratings with employee ratings at the individual or unit level. Therefore, conclusions about direct self–other discrepancies cannot be drawn. Nevertheless, the observed difference between managers' self-ratings and employees' perceptions is consistent with broader evidence of differences in leadership ratings. Research shows that discrepancies between leaders' self-ratings and subordinates' ratings remain common even in recent empirical studies (33, 34). Such overestimation tends to be associated with lower subordinate job satisfaction and greater turnover intention (35), underlining that self–other rating gaps are meaningful and should be considered when interpreting high self-reported leadership scores. In nursing, relational and person-centered leadership frameworks emphasize the importance of leaders actively seeking feedback from non-management staff and engaging in reflexive dialogue about how leadership is experienced at the point of care. Cardiff and colleagues describe person-centered leadership as a dynamic, relational practice that supports mutual trust, empowerment, and well-being, and explicitly call for reflective spaces where leaders and non-management staff can co-interpret their experiences (23). Our finding of a self–other gap therefore highlights the relevance for multi-source feedback and reflective practices when implementing positive leadership models such as PERMA-Lead.

A key result of this study is that PERMA-Lead was associated with lower intention to leave the profession, even after controlling for a broad range of psychosocial and workplace factors. This is consistent with previous work suggesting that leadership is an important resource for nurse retention. Systematic reviews have demonstrated that relational and transformational leadership practices are positively associated with non-management staff nurses' intent to stay, partly through improving the work environment and strengthening professional commitment (36). More recent studies on engaging leadership underline that leaders who inspire, empower, and connect nurses to a shared vision can enhance work engagement and reduce burnout and turnover intentions by increasing job resources and intrinsic motivation (31). Within the Model of causes and consequences of work-related stress, leadership is conceptualized as a job resource that fuels the motivational pathway (engagement, commitment, intention to stay), whereas high job demands and work–home conflict primarily drive the health impairment pathway (burnout and related outcomes). Our results are consistent with this pattern: PERMA-Lead appears particularly relevant for nurses' long-term career decisions, while more proximal health outcomes appear more closely related to structural and psychosocial stressors.

The findings for job satisfaction and burnout symptoms require differentiated interpretation. In the fully adjusted models, job satisfaction was primarily related to core work resources such as social community, rewards, and bond with the organization. However, sensitivity analyses showed that the association between PERMA-Lead and job satisfaction changed across model specifications and became stronger when conceptually overlapping variables were excluded. This indicates that the association with job satisfaction is sensitive to model specification and attenuated in the fully adjusted model, consistent with conceptual overlap and potential overadjustment. Accordingly, this finding should not be interpreted as evidence of the absence of an association, but rather as reflecting uncertainty related to model specification.

In contrast, the findings for burnout symptoms were more stable. PERMA-Lead was not significantly associated with burnout in either the fully adjusted or the parsimonious models, suggesting that this finding is comparatively stable and less affected by model specification. Instead, burnout symptoms were consistently associated with insecurity of the working environment and work–private life conflict. This is consistent with recent evidence that unfavorable work environments, understaffing, and chronic workload are powerful predictors of burnout and turnover intention among nurses (5, 37).

This pattern is also consistent with the theoretical model of work-related stress outlined in the introduction, in which job demands are more closely associated with the health impairment pathway (e.g., burnout), while job resources are more closely associated with motivational processes. From a theoretical perspective, high demands such as quantitative workload, emotional strain, and work–life conflict may be more proximal to burnout symptoms and may be only partially buffered by leadership. Consistent with this interpretation, the association between PERMA-Lead and burnout remained non-significant across model specifications.

Taken together, these findings indicate that the evidence is strongest for an association between PERMA-Lead and intention to leave the profession, whereas findings for job satisfaction are sensitive to model specification and therefore more uncertain, while findings for burnout symptoms appear more stable.

Further, PERMA-Lead showed a strong correlation with leadership quality (*r* = .73), indicating substantial overlap but not complete redundancy. While PERMA-Lead reflects a positive psychology-based leadership approach emphasizing flourishing, meaning, engagement, and strengths, leadership quality as assessed by the COPSOQ captures more functional and task-oriented aspects such as planning, role clarity, and conflict management. This overlap may have affected the interpretation of the PERMA-Lead coefficient and contributed to attenuation in the fully adjusted models, reflecting potential overadjustment.

Our findings suggest that positive leadership may represent an important relational resource within the work environment, but it is unlikely to be sufficient on its own to address burnout or job satisfaction. Leadership approaches grounded in person-centered values may be particularly relevant for relational and motivational resources (38), while burnout and exhaustion are more strongly associated with structural working conditions and workload-related demands. These findings suggest that leadership-focused approaches may benefit from being considered alongside structural measures related to staffing levels, workload, scheduling, and work-life balance.

Conceptually, our results also contribute to the emerging discussion on the alignment between positive leadership and person-centredness in nursing. PERMA-Lead is grounded in the PERMA wellbeing framework and focuses on promoting positive emotions, engagement, relationships, meaning, and accomplishment among employees. Person-centered nursing and person-centered leadership, in turn, aim to create “healthful” relationships and cultures that support human flourishing for patients and staff (21, 23). The PERMA elements map closely onto key outcomes of person-centered practice, such as experiencing meaningful engagement in care, feeling valued and connected, and having opportunities to grow. In this sense, PERMA-Lead may be understood as one possible operationalization of person-centered leadership that translates person-centered values into concrete, observable leadership behaviors. The association with lower intention to leave in our study is consistent with the argument that leadership approaches which integrate flourishing and person-centredness are not only ethically desirable but may be relevant in the context of addressing challenges related to nurse retention.

### 4.1 Strengths & limitations

This study has several strengths that enhance the relevance and interpretability of its findings. It is one of the first studies to apply the PERMA-Lead framework within a nursing context, thereby extending positive leadership research into a field where staff well-being and retention are of critical concern. By capturing both employee and manager perspectives, the study provides a more nuanced and multifaceted understanding of leadership dynamics than single-source assessments typically allow. The use of validated and widely applied COPSOQ scales, in combination with the PERMA-Lead Profiler, supported reliable and valid measurement of both psychosocial work factors and leadership behaviors. Furthermore, the analytic strategy included multiple imputation for missing data, heteroscedasticity-consistent robust standard errors, and the inclusion of a comprehensive set of established workplace predictors based on prior research. This allowed the study to examine the incremental association of positive leadership above and beyond well-known structural and psychosocial factors, thereby situating PERMA-Lead within a complex and ecologically valid work environment.

However, this study has several limitations that should be considered when interpreting the findings. First, the cross-sectional design precludes any causal conclusions about the relationships between positive leadership and health-related outcomes; reverse causation cannot be ruled out, as employees with more favorable attitudes may perceive leadership more positively. Second, all variables were assessed through self-report, which introduces potential response biases, including social desirability. This also raises the possibility of common method variance, which may have inflated observed associations. This is particularly relevant for leadership ratings, as leaders tend to evaluate their own behaviors more favorably than followers do.

Generalizability is further limited by the single-organization design and the 23% response rate, which raises the possibility of non-response bias. It is plausible that more engaged employees or those with more favorable perceptions of leadership were more likely to participate. This may have contributed to inflated PERMA-Lead scores and a more positive overall picture of leadership. In addition, such selective participation may have influenced not only the descriptive findings but also the estimated strength and direction of associations between variables.

Third, participation was voluntary, which may have resulted in a healthier or more motivated subgroup of staff completing the survey. Finally, the modeling strategy included a relatively large number of predictors, some of which are conceptually related to PERMA-Lead (e.g., leadership quality, social support, and meaning of work). While this approach was chosen to assess the incremental contribution of positive leadership beyond established factors, the inclusion of conceptually overlapping variables may have affected the interpretation of the PERMA-Lead coefficient and introduced the possibility of overadjustment. In addition, the number of predictors in relation to the sample size may have reduced estimate stability and contributed to specification-dependent findings. The models were specified to estimate adjusted associations rather than to optimise prediction and should therefore be interpreted with appropriate caution.

Sensitivity analyses indicated that some results, particularly for job satisfaction, varied depending on model specification, whereas others, such as the association with intention to leave the profession and the absence of an association with burnout symptoms, remained more stable. These findings highlight that the interpretation of PERMA-Lead depends in part on the chosen adjustment strategy.

These limitations should be considered, and future research would benefit from more parsimonious modeling approaches, multisource leadership assessments, higher response rates, broader sampling, and longitudinal designs.

## 5 Conclusion

This study indicates that positive leadership, operationalized as PERMA-Lead, is perceived as high among nurses and managers, although differences between management and non-management perspectives were observed. While PERMA-Lead showed no consistent association with burnout symptoms, its association with job satisfaction was sensitive to model specification, with no significant association observed in the fully adjusted model. In contrast, PERMA-Lead was consistently associated with a lower intention to leave the profession, suggesting an association between positive leadership and retention-related attitudes.

Overall, positive leadership may represent a relevant relational resource within the work environment but may be best understood as complementary to, rather than a substitute for, structural improvements in working conditions. Future research could build on these findings to inform the development and evaluation of longitudinal and intervention studies, which are required to test the effectiveness of leadership approaches. Such research may also include multi-source assessments of leadership and further examine the distinct and overlapping contributions of leadership constructs.

## Data Availability

The raw data supporting the conclusions of this article will be made available by the authors, without undue reservation.
