# Supplementary material for: Positive leadership and health-related long-term outcomes among hospital nurses: a cross-sectional study
Source: Front Health Serv. 2026 May 22;6:1789258. doi: 10.3389/frhs.2026.1789258 (PMC13236877; doi:10.3389/frhs.2026.1789258)
Supplement: Supplementary file 2 [file Datasheet2.pdf]

Supplementary File B: Specification of parsimonious models and excluded variables used in sensitivity analyses

The following variables were excluded from the parsimonious models due to their conceptual proximity to PERMA-Lead and the resulting potential for overlap in the measurement of leadership-related and motivational work characteristics.

**Meaning of work** was excluded because it directly reflects the perception of meaningfulness and purpose in work, which represents a core component of the PERMA framework, particularly the dimension of meaning.

**Opportunities for development** were excluded as they capture growth, learning, and personal development at work. These aspects are closely aligned with the PERMA dimensions of engagement and accomplishment, as well as with leadership behaviors that foster employee development.

**Bond with the organization** was excluded because it reflects affective attachment and identification with the organization, which are theoretically linked to relational and motivational processes influenced by leadership.

**Feedback** was excluded as it represents a core leadership behavior related to recognition, guidance, and performance support. Such behaviors overlap with the positive, strengths-based leadership practices captured by PERMA-Lead, particularly in fostering accomplishment and engagement.

**Quality of leadership** was excluded due to its strong conceptual and empirical overlap with PERMA-Lead. While assessed in more functional terms (e.g., planning, role clarity, conflict management), it nevertheless captures central aspects of leadership behavior.

**Social community at work** and **social support at work** were excluded because they reflect relational aspects of the work environment that are closely shaped by leadership. PERMA-Lead explicitly emphasizes positive relationships, and leadership behavior is a key driver of team climate and perceived support.

**Rewards** were excluded as they capture recognition, appreciation, and perceived fairness of compensation or acknowledgment. These aspects are closely linked to leadership practices that foster motivation and appreciation.
