# Supplementary material for: Positive leadership and health-related long-term outcomes among hospital nurses: a cross-sectional study
Source: Front Health Serv. 2026 May 22;6:1789258. doi: 10.3389/frhs.2026.1789258 (PMC13236877; doi:10.3389/frhs.2026.1789258)
Supplement: Supplementary file 3 [file Datasheet3.pdf]

Supplementary file C: Proportion of missing data for all variables included in the analyses

Variable	Missing n	Missing %
PERMA-Lead	0	0.0
Age	4	1.9
Years of professional experience	7	3.3
Sex	0	0.0
Emotional demands	14	6.6
Physical demands	13	6.2
Quantitative demands	11	5.2
Demands to hide emotions	31	14.7
Meaning of work	12	5.7
Influence at work	17	8.1
Opportunities for development	11	5.2
Scope for breaks/holidays	30	14.2
Bond with the organization	30	14.2
Feedback	27	12.8
Quality of leadership	23	10.9
Social community at work	25	11.8
Role conflict	19	9.0
Social support at work	24	11.4
Unfair behaviour	30	14.2
Insecurity of the working environment	16	7.6
Rewards	23	10.9
Difficulties with demarcation	27	12.8
Work–private life conflict	26	12.3
Shift work	23	10.9
Intention to leave the profession	29	13.7
Job satisfaction	30	14.2
Burnout symptoms	29	13.7
