# Supplementary material for: Positive leadership and health-related long-term outcomes among hospital nurses: a cross-sectional study
Source: Front Health Serv. 2026 May 22;6:1789258. doi: 10.3389/frhs.2026.1789258 (PMC13236877; doi:10.3389/frhs.2026.1789258)
Supplement: Supplementary file 5 [file Datasheet5.pdf]

Supplementary file E: Sensitivity analyses using parsimonious regression models excluding conceptually proximal variables.

| | Intention to leave the profession | | | | Job satisfaction | | | | Burnout-Symptoms | | | |
|---|---|---|---|---|---|---|---|---|---|---|---|---|
| **Predictor** | **β** | **SE** | **95% CI** | **p** | **β** | **SE** | **95% CI** | **p** | **β** | **SE** | **95% CI** | **p** |
| Intercept | 62.71 | 20.35 | 22.32 – 103.10 | .003 | 70.66 | 7.90 | 54.92 – 86.39 | .001 | 5.01 | 16.73 | -28.29 – 38.30 | .766 |
| PERMA-Lead | -0.36 | 0.15 | -0.67 – -0.05 | .024 | 0.24 | 0.06 | 0.12 – 0.36 | .001 | 0.20 | 0.14 | -0.07 – 0.47 | .152 |
| Age | -0.26 | 0.34 | -0.93 – 0.41 | .438 | | | | | -0.08 | 0.15 | -0.38 – 0.22 | .612 |
| Years of professional experience | -0.05 | 0.36 | -0.75 – 0.65 | .888 | -0.07 | 0.07 | -0.21 – 0.08 | .356 | | | | |
| Sex: Male | -6.20 | 6.10 | -18.29 – 5.89 | .312 | | | | | -16.00 | 5.40 | -26.70 – -5.30 | .004 |
| Emotional demands | 0.07 | 0.15 | -0.23 – 0.37 | .650 | 0.00 | 0.06 | -0.11 – 0.12 | .970 | | | | |
| Quantitative demands | 0.05 | 0.12 | -0.19 – 0.30 | .677 | -0.05 | 0.05 | -0.16 – 0.06 | .382 | | | | |
| Physical demands | | | | | -0.06 | 0.04 | -0.14 – 0.02 | | 0.12 | 0.08 | -0.03 – 0.28 | .145 |
| Demands to hide emotions | 0.10 | 0.10 | -0.10 – 0.29 | .317 | -0.06 | 0.04 | -0.14 – 0.01 | .097 | 0.15 | 0.08 | -0.01 – 0.31 | .070 |
| Influence at work | -0.31 | 0.11 | -0.54 – -0.09 | .008 | 0.03 | 0.04 | -0.05 – 0.12 | .418 | -0.01 | 0.10 | -0.21 – 0.18 | .880 |
| Scope for breaks/holidays | | | | | | | | | 0.07 | 0.09 | -010 – 0.25 | .412 |
| Role conflict | -0.05 | 0.10 | -0.25 – 0.16 | .650 | -0.04 | 0.04 | -0.12 – 0.04 | .298 | | | | |
| Unfair behaviour | -0.03 | 0.09 | -0.21 – 0.14 | .712 | 0.01 | 0.04 | -0.07 – 0.09 | .774 | 0.02 | 0.08 | -0.15 – 0.18 | .829 |
| Insecurity of the working environment | 0.18 | 0.08 | 0.19 – 0.34 | .028 | -0.14 | 0.03 | -0.21 – -0.07 | .001 | 0.18 | 0.07 | 0.03 – 0.33 | .018 |
| Difficulties with demarcation | -0.03 | 0.10 | -0.23 – 0.16 | .738 | | | | | -0.11 | 0.09 | -0.29 – 0.07 | .222 |
| Work–private life conflict | 0.18 | 0.12 | -0.06 – 0.42 | .145 | -0.11 | 0.04 | -0.13 – -0.03 | .008 | 0.47 | 0.10 | 0.27 – 0.67 | < .001 |
| Shift work | -7.36 | 4.87 | -17.03 – 2.30 | .134 | | | | | | | | |

Regression coefficients (β), standard errors (SE), confidence intervals (CI), and p-values from sensitivity analyses using parsimonious models excluding conceptually proximal variables. Estimates are based on pooled results from multiple imputation (m = 20).
